# Acute myeloid leukemia with t(7;21)(p22;q22) and 5q deletion: a case report and literature review

**DOI:** 10.1186/2162-3619-3-8

**Published:** 2014-03-19

**Authors:** Jianling Ji, Eric Loo, Sheeja Pullarkat, Lynn Yang, Carlos A Tirado

**Affiliations:** 1 Cytogenetics, Department of Pathology & Laboratory Medicine, David Geffen School of Medicine at UCLA, UCLA, Los Angeles, CA, USA; 2 Hematopathology, Department of Pathology & Laboratory Medicine, David Geffen School of Medicine at UCLA, UCLA, Los Angeles, CA, USA

**Keywords:** AML, Acute myeloid leukemia, t(7;21), t(7;21)(p22;q22), *RUNX1*, *USP42*

## Abstract

The gene *RUNX1* at chromosome 21q22 encodes the alpha subunit of Core binding factor (CBF), a heterodimeric transcription factor involved in the development of normal hematopoiesis. Translocations of *RUNX1* are seen in several types of leukemia with at least 21 identified partner genes. The cryptic t(7;21)(p22;q22) rearrangement involving the *USP42* gene appears to be a specific and recurrent cytogenetic abnormality. Eight of the 9 cases identified in the literature with this translocation were associated with acute myeloid leukemia (AML), with the remaining case showing refractory anemia with excess blasts, type 2. Herein, we present a patient with two preceding years of leukopenia and one year of anemia prior to the diagnosis of AML, NOS with monocytic differentiation (myelomonocytic leukemia) whose conventional cytogenetics showed an abnormal clone with 5q deletion. Interphase FISH using LSI *RUNX1/RUNXT1* showed three signals for *RUNX1*. FISH studies on previously G-banded metaphases showed the extra *RUNX1* signal on the short arm of chromosome 7. Further characterization using the subtelomeric 7p probe showed a cryptic 7;21 translocation. Our case and eight previously reported leukemic cases with the t(7;21)(p22;q22) appear to share similar features including monocytic differentiation, immunophenotypic aberrancies (often with CD56 and/or CD7), and a generally poor response to standard induction chemotherapy. About 80% of these cases had loss of 5q material as an additional abnormality at initial diagnosis or relapse. These findings suggest that t(7;21) may represent a distinct recurrent cytogenetic abnormality associated with AML. The association between the t(7;21) and 5q aberrancies appears to be non-random, however the pathogenetic connection remains unclear. Additional studies to evaluate for *RUNX1* partner genes may be considered for AML patients with *RUNX1* rearrangement and 5q abnormalities; however knowledge of the prognostic implications of this rearrangement is still limited.

## Introduction

Gene fusion and chromosomal abnormalities play vital roles in tumorigenesis, including hematologic malignancies [1]. More than 100 fusion genes have been reported in acute myeloid leukemia (AML) [2]. Many of these fusion genes involve the translocation of Runt-related transcription factor 1 (*RUNX1)*, also known as acute myeloid leukemia 1 protein (*AML1*) [3]. The gene *RUNX1* at chromosome 21q22 encodes the alpha subunit of core binding factor (CBF), a heterodimeric transcription factor involved in the development of normal hematopoiesis. At least 21 partner genes of *RUNX1* have been identified. Translocations of *RUNX1* have been reported in acute lymphoblastic leukemia (ALL) [4,5], acute myeloid leukemia (AML), and myelodysplastic syndromes (MDS). In AML, the t(8;21)(q22;q22) is the most frequent translocation involving *RUNX1*. In contrast, the much rare t(7;21)(p22;q22) involving a rearrangement with the *USP42* gene, is cryptic, but appears to be a specific and recurrent cytogenetic abnormality.

The deletion of all or part of long arm of chromosome 5 (del(5q)) is a recurrent abnormality in AML and MDS patients. While the association between del(5q) and AML are still unclear, a deletion of 1.5 Mb region at 5q31.1 flanked by genes *IL-9* and *EGR-1* has been reported to be related with AML [6]. In addition, del(5q) is mostly part of the complex karyotype in many AML cases, which makes it more difficult to reveal its significance.

In this study, we describe a new AML case with a cryptic t(7;21)(p22;q22) and chromosome 5q abnormality. The case reported here shares the common features with the few other cases reported in the literature, suggesting that the t(7;21)(p22;q22) may represent a distinct entity in AML.

## Clinical presentation

The patient is a 57-year-old female who was referred for hematology consult after presenting with a two-year history of chronic leukopenia with progression to include anemia of several months duration. A bone marrow biopsy was performed, revealing approximately 24% myeloblasts by immunohistochemistry, with the blasts expressing monocytic differentiation with aberrant CD56 expression by flow cytometry. She was diagnosed with AML, and was referred to an academic center for inpatient chemotherapy and possible transplant. Following evaluation, she was placed on Revlimid therapy as opposed to induction chemotherapy for about 5 months without improvement or significant deterioration of her blood counts. She was referred to our institution to be evaluated for allogenic stem-cell transplantation. A repeat bone marrow biopsy (about 6–7 months post diagnosis) confirmed persistent AML, and the patient was started on 7 + 3 AML induction (Cytarabine 320 mg IV continuous days 1–7, Idarubicin 19 mg IV on days 3–6). A day 16 repeat bone marrow biopsy showed persistent presence of abnormal myeloblasts. Biopsy about 6 weeks following induction therapy showed remission with no excess or abnormal myeloblasts. She completed consolidation chemotherapy with high-dose cytarabine (3 gm/m2 IV q 12 hours on days 1, 3, 5 for 6 doses), and recently had a successful allogenic stem-cell transplant.

## Materials and methods

### Histologic methods

Particle smears and touch preparations were prepared upon receipt of specimen, air dried, and stained with Wright-Giemsa. Trephine biopsy and aspirate clot sections were fixed in B5 and buffered formalin, respectively. Following fixation, tissues were paraffin-embedded, and 4-μm thick sections were cut and stained with hematoxylin-eosin.

### Flow cytometry

A WBC suspension is created from the bone marrow aspirate sample using bulk lysing with BD Pharmlyse (ammonium chloride base) and PBS wash. 6-Color custom cocktails (FITC/PE/PerCPCy5.5/APC/APCH7/V450 fluorochrome format) for immunophenotyping were manufactured by the CTT group of Becton Dickinson. The antibody staining and fixation include a 15 minute incubation in the dark, PBS washing, and resuspension & fixation with 1% paraformaldehyde. The Becton Dickinson FACSCanto II (3-laser) flow cytometer with BD FACSDiva Software were used for event acquisition, and FCS Express Version 3.00.0821 (DeNovo Software, Los Angeles, CA) was used for analysis.

### Cytogenetic studies

Conventional chromosome analysis was performed on bone marrow cells using standard cytogenetic techniques and G-banding. A total of 20 metaphase cells were analyzed. Karyotypes were prepared using Applied Imaging CytoVision software and described according to the guidelines of International System for Human Cytogenetic Nomenclature (ISCN, 2013) [7].

### Fluorescence in situ hybridization (FISH)

FISH studies were performed on both interphase nuclei and previously G-banded metaphases from the patient’s bone marrow sample. Interphase FISH probes included: EGR1/D5S23-D5S721, D7S486 (7q31)/CEP 7, CEP 8 (D8Z2)/D20S108 (20q12), *PML/RARA* Dual Color (DC) Dual Fusion (DF), *BCR/ABL* DC, DF, EVI1 Tri-color (3q26), CBFB DC (16q22), *MLL* DC (11q23) Break Apart and *RUNX1*T1/*RUNX1* (ETO/AML1) DC DF probe. Metaphase FISH on previously G-banded slides included *RUNX1*T1/*RUNX1* (ETO/AML1) DC DF probe, TelVysion 7p, 7q and 14q probe (subtelomeric cocktail). All the probes were from Abbott Molecular (Des Plaines, Illinois 60018).

## Results

### Morphology and immunophenotypic findings

**Figure 1:**
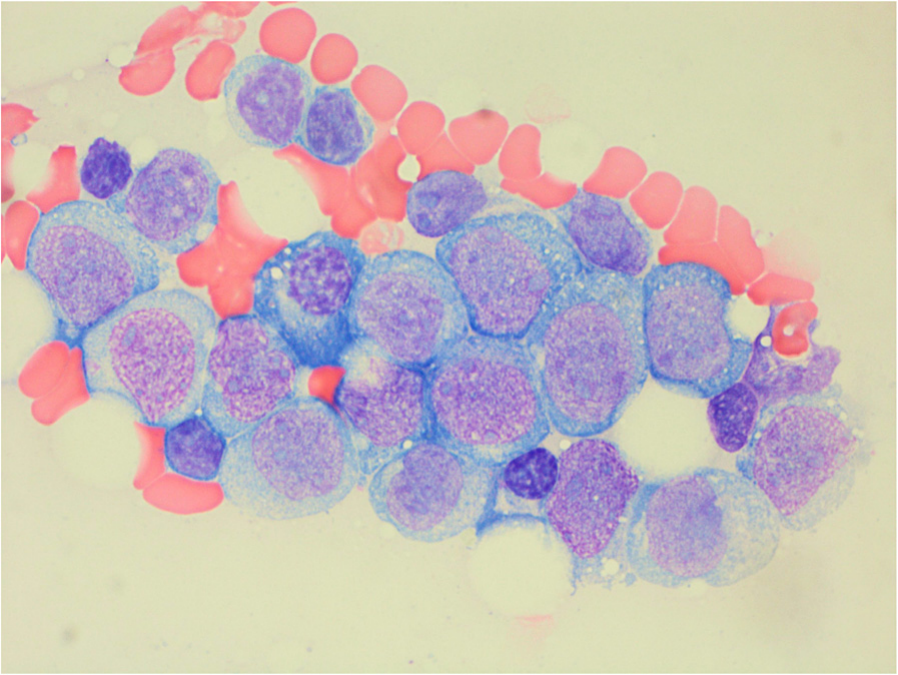
**Aspirate smear, Wright-Giemsa, 100× – cluster of intermediate-sized blasts with irregular nuclear contours, fine chromatin with 1–2 nucleoli, and relatively abundant cytoplasm with occasional small vacuoles and rare azurophilic granules.** Rare small lymphocytes and plasma cells are intermixed.

**Figure 2:**
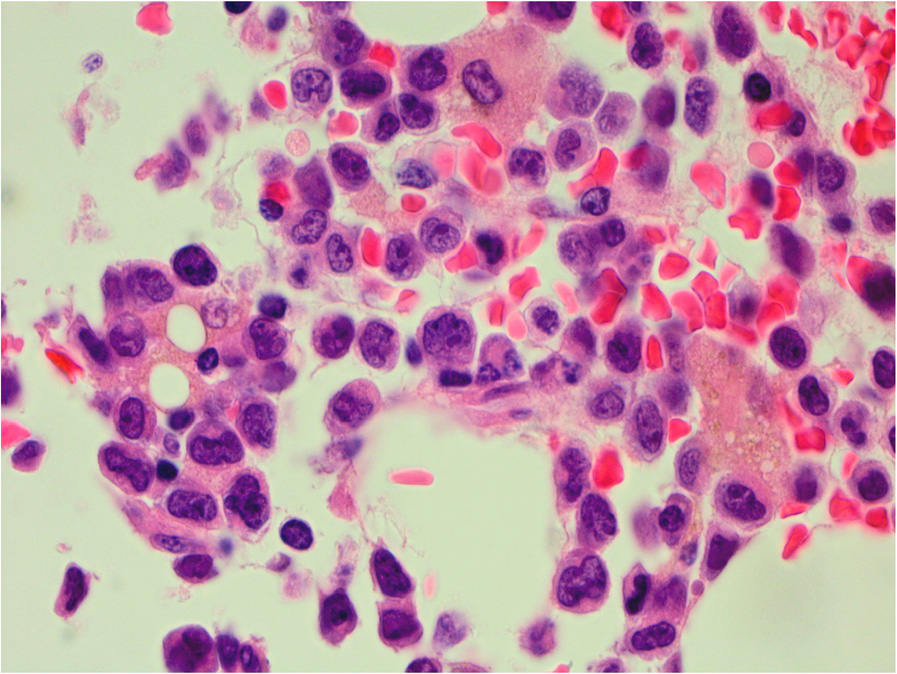
Trephine biopsy, Hematoxylin & Eosin, 100× – Cluster of blasts with background small lymphocytes, plasma cells, and histiocytes.

**Figure 3:**
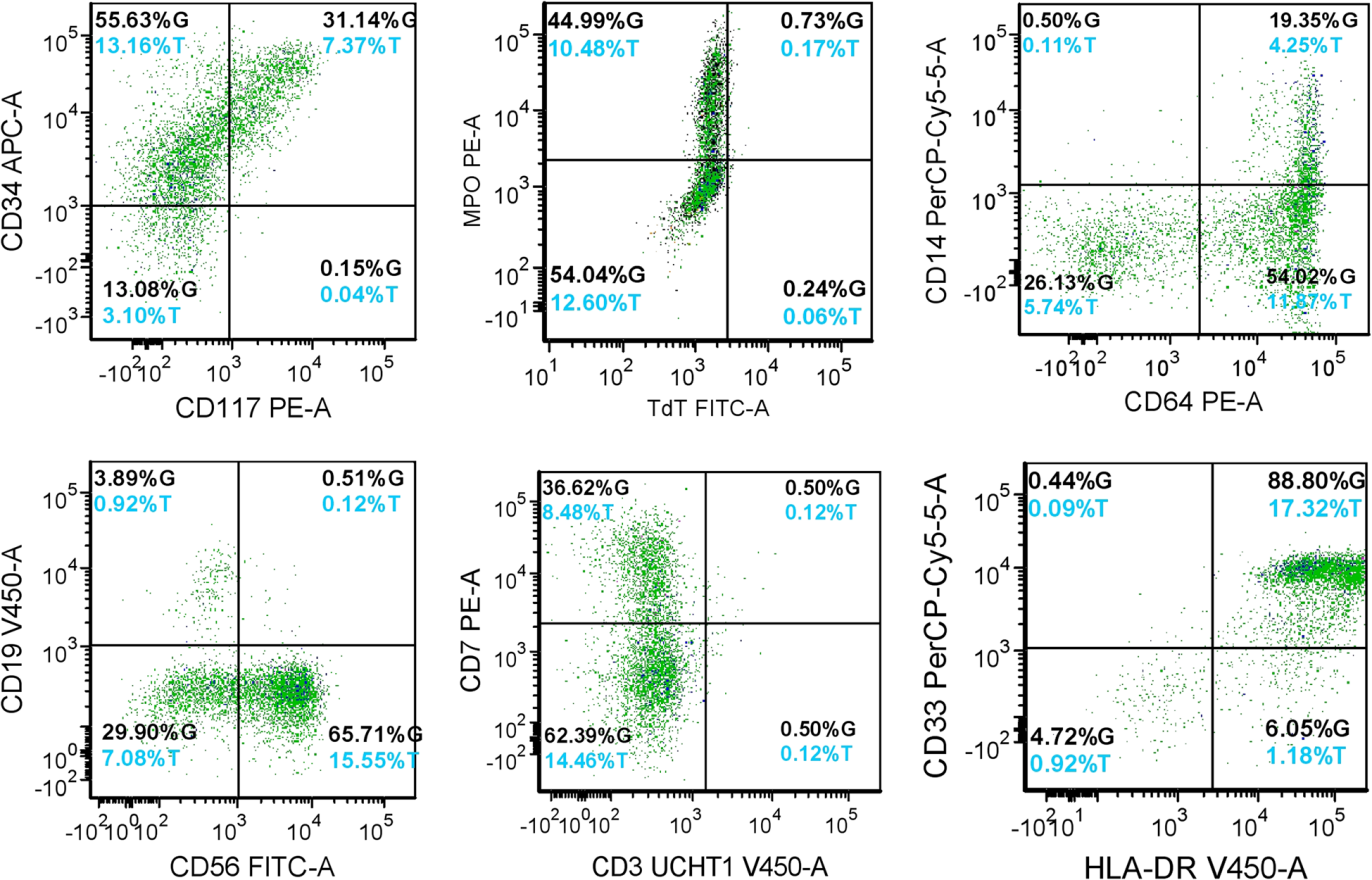
Blast immunophenotype, flow cytometry histograms (closed gate) – Note expression of myeloid and monocytic immunophenotypic markers with aberrant expression of CD7 and CD56.

The bone marrow biopsy demonstrated a hypocellular marrow with increased blasts representing about 25% of the cellular marrow elements (Figures 1 and 2). Background hematopoiesis was suppressed and left-shifted, but did not show morphologic evidence of dysplasia. Immunophenotyping of the abnormal blasts by flow cytometry (Figure 3) showed expression of CD10 (partial), CD11b (partial), CD13, CD14 (partial), CD15 (partial), CD16 (partial), intracytoplasmic CD22 (partial), surface CD22 (partial), CD34 (partial), CD36 (partial), CD38, CD56 (partial), CD64 (partial), CD117 (partial), HLA-DR (bright), and Myeloperoxidase (partial). Features of myelomonocytic dysmaturation were present.

### Cytogenetic analysis

**Figure 4:**
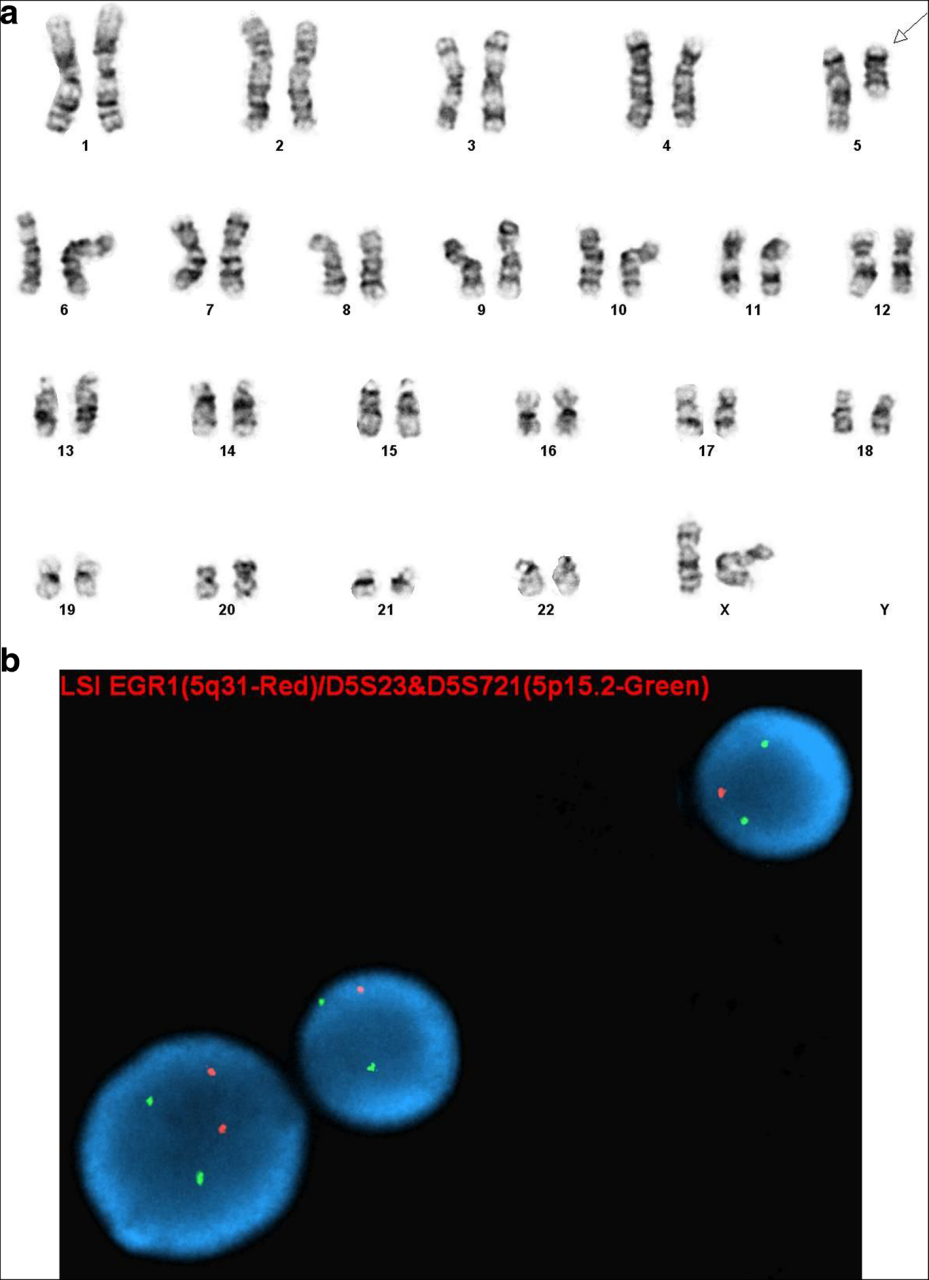
**(a) Karyotype on the bone marrow aspirate showing an additional material of unknown origin attached on 5q leading to 5q loss. (b)** Interphase FISH with two signals of 5p15.2 region (green) and one signal of *EGR1* (orange) suggests loss of 5q.

Chromosome analysis of the bone marrow revealed additional material of unknown origin at 5q13. This unbalanced rearrangement characterized as “add” (ISCN, 2013) led to partial deletion of 5q in 5 of the 20 metaphase cells examined. The initial karyotype was described as: 46,XX,add(5)(q13) [5]/46,XX[15] (Figure 4a). This finding was confirmed by the concurrent interphase FISH study (Figure 4b).

## FISH

**Figure 5:**
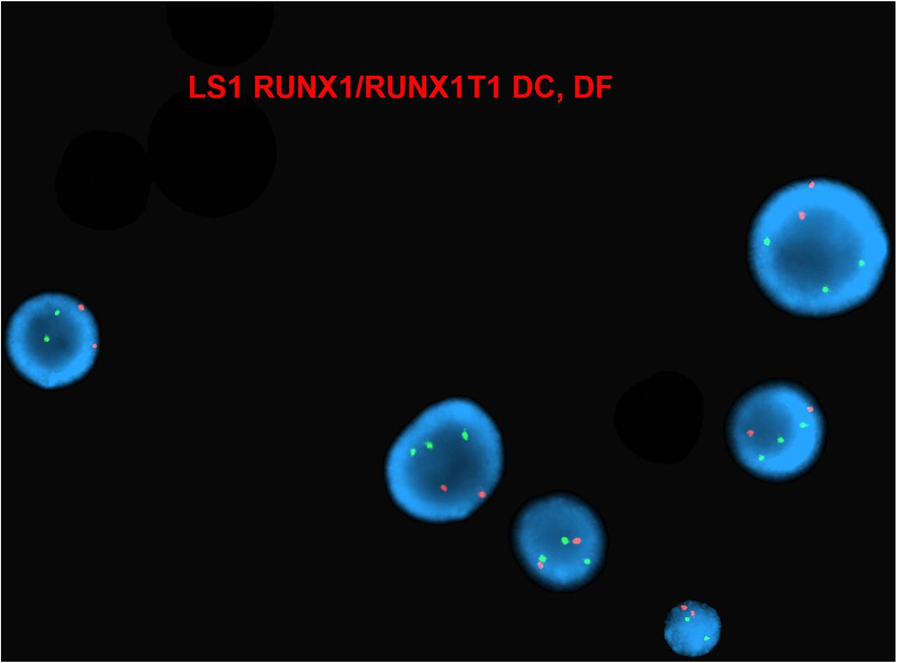
**Interphase FISH with *RUNX1/RUNXT1 *** **dual color dual fusion probe showed three signals of *RUNX1 *** **(green) and two signals of *RUNXT1 *** **(orange).**

**Figure 6:**
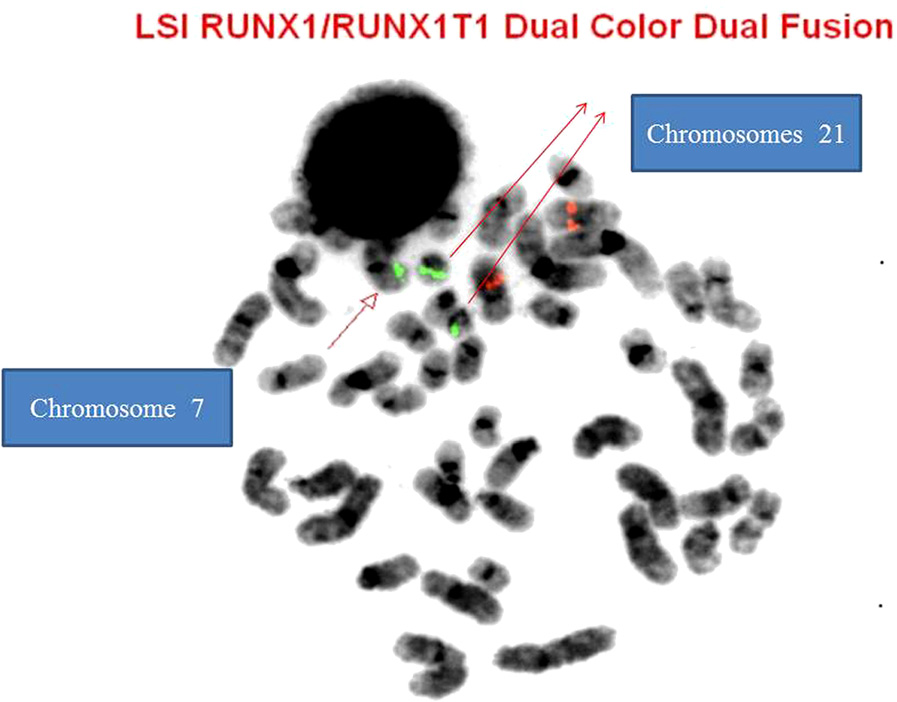
**FISH with LSI *RUNX1/RUNXT1 *probe showing the green signals of *RUNX1 *on der(7), der(21) and normal chromosome 21, respectively.** Two normal orange of *RUNXT1* are seen on the chromosomes 8.

**Figure 7:**
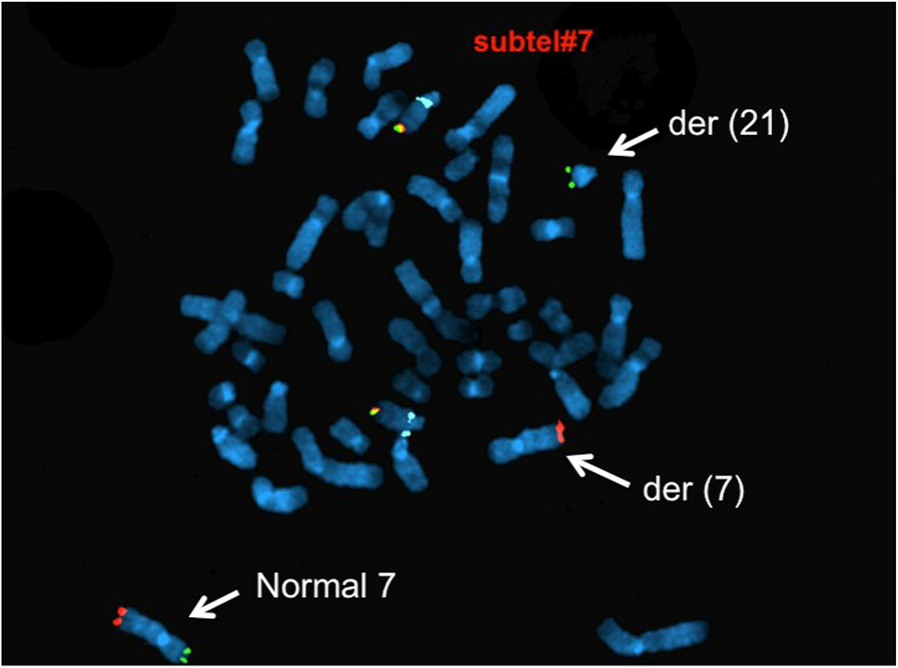
**FISH with TelVysion 7p (green, on the sub-telomere region of 7p), 7q (orange, on the sub-telomere region of 7q) and chromosome 14 (yellow and aqua) showing one green signal of 7p on der(21) and the der(7) is missing a green signal.** Two normal chromosomes 14 are seen as indicated by the signal pattern (one yellow and one aqua signals on two normal chromosomes 14 respectively).

The *RUNX1/RUNXT1* DC DF interphase FISH exhibited three green signals for *RUNX1* (Figure 5). FISH studies on previously G-banded metaphases showed that the extra *RUNX1* signal was on the short arm of chromosome 7 (Figure 6). Further characterization using the subtelomeric probe (TelVysion 7p, 7q and 14q cocktail) confirmed a cryptic 7;21 translocation (Figure 7). Based on the metaphase FISH study, the final karyotype of the bone marrow was characterized as: 46,XX,add(5)(q13)[5]/46,XX[15].ish t(7;21)(p22;q22)(*RUNX1*+; VIJyRM2185+).

## Discussion

In this study, we describe a new case of semi-cryptic t(7;21)(p22;q22) involving the *RUNX1* gene and the loss of chromosome 5q in an AML patient. First reported by Paulsson et al. in AML [8], only 9 cases of t(7;21)(p22;q22) have been reported in to date (Table 1) [2,8-12]. Literature review shows that t(7;21) is a recurrent cytogenetic abnormality in AML [9-11]. The incidence rate of this abnormality varies among reports but is relatively low; Giguere et al. [2] reported approximately 1% in 95 AML patients, and Jeandidier et al. reported 0.75% in 397 cases of AML.

**Table 1:** Reported cases with t(7;21)(p22;q22)

**Report**	**Case**	**Diagnosis/Subtype**	**Age/Sex**	**Flow aberrancies**	**Induction regimen**	**Remission post-induction**	**5q abnormalities**	**Outcome**
		*AML, NOS*						
Index case	1	Monocytic	56 yo, F	CD7, CD56	Cytarabine/Idarubicin “7+3”	Yes	Yes	CR 51 days s/p induction. Completed consolidation and recently underwent unrelated SCT
Paulsson, et al. [8]	2	Minimal differentiation	7 yo, M	Partial CD56	NOPHO-AML-93 protocol	No	Yes (at relapse)	Relapse at 5 years with successful re-induction
Foster, et al. [9]	3	Monoblastic/Monocytic	68 yo, M	NA	MidAC		No	Dead at 5 years from unrelated illness
Giguère, et al. [2]	4	Without maturation	32 yo, M	CD7, CD56	Cytarabine/Daunorubicin	NA	Yes	Died of fungal infection within months of dx
Jeandidier, et al. [10]	5	Myelomonocytic vs. Monblastic/Monocytic	54 yo, M	CD2, CD7, CD22, CD56	GOELAMS	No	Yes	Relapse at 23 months, DOD
	6	Monoblastic/Monocytic	33 yo, M	CD7, CD56	SHG AML96	No	Yes	CR after salvage chemo and allogenic transplant, alive at 36 months
	7	Myelomonocytic	39 yo, M	CD22, CD56	Cytarabine/Idarubicin	No	Yes	Partial response to salvage chemo, dead of bacterial sepsis within months of dx
Gindina, et al. [12]	8	Myelomonocytic	13 yo, M	CD7, CD56, CD71	AIE/HAM	No	Yes	Remission after consolidation chemo, No long-term survival data reported
Panagopoulos, et al. [11]	9	Without maturation	52 yo, F	CD7, CD56	“Standard induction”	NA	Yes	In remission at nine months post dx, no long-term survival data reported
		***MDS***						
Foster, et al. [9]	10	RAEB-2 (17% blasts)	68 yo, F	NA	Mylotarg, Daunorubici, Clofarabine	Yes	No	Remained in remission post maintenance chemotherapy

*Abbreviations: NA* not available, *MIDAC* Mitoxantrone and cytarabine, *GOELAMS* groupe ouest-est des Leucémies et Autres Maladies du Sang 2001 AML protocol, *DOD* died of disease, *SHG AML96* süddeutsche hämoblastose-gruppe protocol, *CR* complete remission, *AIE* cytarabine idarubicin etoposide, *HAM* high dose cytarabine and mitoxantrone.

We compared our case with the other reported t(7;21)(p22;q22) cases (Table 1) [2,8-11]. Of all the 9 reported t(7;21)(p22;q22) cases, eight were diagnosed as AML, and one as an MDS (RAEB-2). In all reported AML patients with t(7;21)(p22;q22) including our current case, the common features in these patients include: 1) Aberrant CD56 expression (8/9 patients) and aberrant CD7 expression (6/9 patients). No patients had expression of CD19, which is commonly seen in t(8;21) [13,14]. 2) Myelomonocytic or monocytic differentiation was common (6/9), and 3/9 patients did not show maturation or showed minimal differentiation. 3) Patients responded poorly to the standard induction chemotherapy. 4) Eight of the 9 patients had loss of chromosome 5q at the initial diagnosis or at relapse.

CD56 expression is seen in about 25% [15,16] of AML cases with t(8;21) (q22;q22), and is generally associated with poor prognosis [16]. Meanwhile, CD7 is expressed in about 30% of AML cases and its positivity is linked with poor prognosis in myeloid malignancies [17]. The t(7;21)(p22;q22) AML cases had CD56 and CD7 positive rates higher than what has been reported in general AML cases, and these patients had an overall poor response to standard chemotherapy (Table 1).

One of the genes located on chromosome 5q is ribosomal protein S14 (*RPS14*), which is essential for the maintenance of normal erythroid progenitor cells [10]. Loss of chromosome 5q causes haploinsufficiency of *RPS14*[18]. Boultwood et al. [19] hypothesized that this haploinsufficiency may be related with failure of erythropoiesis. In our case report, the patient had a history of anemia for one year, and the karyotype showed loss of chromosome 5q. We speculate that the anemia in this patient is related with haploinsufficiency of *RPS14*, however, this speculation would need to be validated by further molecular testing.

As previously noted, deletion of 5q is a common cytogenetic abnormalities in 8 of the 9 patients with t(7;21)(p22;q22). The high incidence of t(7;21)(p22;q22) found together with del(5q) suggest that this combination is a non-random abnormality. Many questions need to be answered, including: Why does this combination happen with such a high frequency? What is the etiology? What is the significance of these aberrancies in relation to AML? Although a recent large scale cooperative study of 514 MDS patients showed that 5q deletion with additional cytogenetic abnormalities is a marker of poor prognosis [20], additional study is needed for this combination in AML. While we cannot provide further experimental evidence, we think one possibility is that two (or more) functionally redundant genes were disrupted by the t(7;21)(p22;q22) and del(5q) respectively. Subsequently, the affected cells would have lost important functions associated with drug resistance, apoptosis, and/or DNA repair.

All previous reports about the t(7;21)(p22;q22) rearrangement have detected a *RUNX1*-*USP42* fusion protein. It is reasonable to assume that our case also has the associated *RUNX1*-*USP42* fusion protein, although we did not pursue the exact partner of *RUNX1* in this report due to the limited specimen. *RUNX1* is a vital regulator of hemopoiesis [21]. Various hemopoietic signaling molecules, growth factors, and transcription factors are regulated by *RUNX1*[22]. *RUNX1* translocation is a recurrent feature in many AML and MDS patients, and numerous translocation partners have been described. *RUNX1* rearrangment’s leukemogenicity lies in the retention of *RUNX1*’s DNA binding “Runt homology domain” (RHD) domain, and associated loss of its transactivation domain. The resultant fusion protein acts as a dominant negative inhibitor of wild-type *RUNX1* in transcription activation [11,22].

While most studies focus on *RUNX1*, ubiquitin specific peptidase 42 (*USP42*) may also play an important leukemogenic role in t(7;21)(p22;q22) cases. *USP42*, belonging to the ubiquitin-proteasome system (UPS), is a deubiquitinating enzyme (DUB). The process of ubiquitination and deubiquitination decides the fate of many proteins, many of which are related with cell cycle regulation, DNA repair, and differentiation. *USP* and the DUBs contain many potential drug targets for the treatment. *USP42* itself was reported to increase the stability of *p53*[23]. We propose that *RUNX1*-*USP42* fusion protein loses wild type *USP42* function and decreases the stability of *p53*. Further studies to characterize the involvement of *USP42* in our case were not possible due to insufficient sample.

In conclusion, we report a new case of AML with a cryptic t(7;21)(p22;q22) and chromosome 5q abnormality. To the best of our knowledge, this represents one of the few AML patients with this genetic aberrancy. Review of the literature showed that our case shares many common features with other reported cases, which suggests that t(7;21)(p22;q22) may represent a new distinct entity in AML.

## Consent

Consent for publication of this case report and any accompanying images was obtained from the patient.

## Competing interests

The authors declare that they have no competing interests.

## Authors’ contributions

JJ and EL contributed equally to get clinical information, flow cytometry and morphological data as well as the cytogenetic and molecular cytogenetic data. LY did all the bench work in this case as well the interpretation of the results. CAT drafted and checked all the manuscript and this case presentation. All authors read and approved the final manuscript.
